# Rapid bacteriolysis of *Staphylococcus aureus* by lysin exebacase

**DOI:** 10.1128/spectrum.01906-23

**Published:** 2023-08-10

**Authors:** Xavier Vila-Farres, Karen Sauve, Jun Oh, Steven Swift, Boudewijn DeJonge, Jane E. Ambler, Raymond Schuch

**Affiliations:** 1 ContraFect Corporation, Yonkers, New York, USA; 2 Janssen Pharmaceuticals, Brisbane, California, USA; 3 Shionogi Inc, Florham Park, New Jersey, USA; University of California, San Diego, La Jolla, California, USA

**Keywords:** lysin, exebacase, direct lytic agent, bacteriolysis, *Staphylococcus aureus*

## Abstract

Lysins (peptidoglycan hydrolases) are promising new protein-based antimicrobial candidates under development to address rising antibiotic resistance encountered among pathogenic bacteria. Exebacase is an antistaphylococcal lysin and the first member of the lysin class to have entered clinical trials in the United States. In this study, the bacteriolytic activity of exebacase was characterized with time-kill assays, turbidity reduction assays, and microscopy. Three methicillin-susceptible *Staphylococcus aureus* and three methicillin-resistant *S. aureus* isolates were tested in time-kill assays over a range of concentrations from 0.25 to 8 × MIC. Exebacase demonstrated a concentration-dependent killing and showed bactericidal activity (≥3 log_10_ kill achieved relative to the starting inoculum) within 3 h at 1 × MIC against all strains tested. Dose-dependent lysis by exebacase was, furthermore, observed in the turbidity reduction assay, wherein decreases in initial OD_600_ of 50% were observed within ~15 min at concentrations as low as 4 µg/mL. Membrane dissolution, loss of cytoplasmic material, and lysis were confirmed by video and electron microscopy. The demonstrated rapid bacteriolytic effect of exebacase is an important distinguishing feature of this novel modality.

**IMPORTANCE:**

To guide the development of an investigational new antibacterial entity, microbiological data are required to evaluate the killing kinetics against target organism(s). Exebacase is a lysin (peptidoglycan hydrolase) that represents a novel antimicrobial modality based on degradation of the cell wall of *Staphylococcus aureus*. Killing by exebacase was determined in multiple assay formats including time-kill assays, wherein reductions of viability of ≥3 log_10_ colony-forming units/mL were observed within 3 h for multiple different isolates tested, consistent with very rapid bactericidal activity. Rapid reductions in optical density were likewise observed in exebacase-treated cultures, which were visually consistent with microscopic observations of rapid lysis. Overall, exebacase provides a novel antimicrobial modality against *S. aureus*, characterized by a rapid cidal and lytic activity.

## INTRODUCTION

Lysins (cell wall hydrolytic enzymes) are a novel class of protein-based antimicrobials currently in clinical development for the treatment of infections with antibiotic-resistant bacterial pathogens (1). Exebacase (CF-301) is a lysin with potent activity against vegetative and biofilm forms of methicillin-susceptible and methicillin-resistant *Staphylococcus aureus* (MSSA and MRSA, respectively) and multidrug-resistant phenotypes (2 – 6). Other important microbiological attributes of exebacase include synergy with other antistaphylococcal antibiotics, the absence of cross-resistance with marketed antibiotics, an extended post-antibiotic effect, and a low propensity for resistance development (2, 6 – 8). These attributes are commonly reported for other lysins (9) and support the development of lysins as a novel antimicrobial modalities.

Lysins act by cleaving specific bonds in the peptidoglycan (murein) sacculus, which protects the cell from osmotic pressure, and this cleavage of peptidoglycan can result in bacteriolysis, in a process generally understood to occur rapidly (10). A rapid bactericidal activity for exebacase against staphylococci was initially reported in early development (prior to first-in-human studies) using cation-adjusted Mueller-Hinton broth (CAMHB) (2). To advance into the clinic, it was necessary to develop an accurate and reproducible method for exebacase MIC determination. For this, a modified broth microdilution (BMD) method was developed based on the use of CAMHB supplemented with horse serum (25%, vol/vol) and 0.5 mM dl-dithiothreitol (CAMHB-HSD) (11). The modified method was reviewed by the Clinical and Laboratory Standards Institute (CLSI) Methods Development and Standardization Working Group and expected quality control (QC) ranges for *Staphylococcus aureus* American Type Culture Collection (ATCC) 29213 and *Enterococcus faecalis* ATCC 29212 established using CAMHB-HSD were reviewed by the QC working group; the method was subsequently approved by the CLSI Subcommittee on antimicrobial susceptibility testing (AST) and detailed in CLSI document M100 (12). Exebacase is the first protein-based direct lytic agent (e.g., a nonantibiotic) to be reviewed by a recognized standards development organization (CLSI).

With a standardized AST method established by CLSI, it was necessary to assess the performance characteristics of the method and conduct studies to address the influence of the growth medium (e.g., pH, divalent cations, age of medium), inoculum density, and incubation conditions, such as atmospheric conditions and duration of incubation (11). Furthermore, more recently, special instructions for reading exebacase MIC endpoints including photographs are detailed in CLSI M100 (12). The use of standardized methods and concomitant QC testing provides confidence to the *in vitro* data generated when testing target bacteria when a modified method is adopted as a standard method for the testing of clinical isolates all prior *in vitro* testing needs to be retested using the standard medium. In this report, time-kill assays were performed with several MSSA and MRSA isolates at multiples of the exebacase MIC in CAMHB-HSD. Rapid bactericidal activity was demonstrated and then, furthermore, linked to bacteriolytic activity determined using an optical density-based assay and to the direct visualization of such activity by microscopy. The findings demonstrate a rapid bacteriocidal and bacteriolytic effect of exebacase on *S. aureus*, consistent with its perceived mode of action.

## MATERIALS AND METHODS

### Strains, reagents, and culture conditions

The following reagents were provided by the Network on Antimicrobial Resistance in *Staphylococcus aureus* (NARSA) for distribution by BEI Resources, NIAID, NIH: MRSA Strain NRS123 (MW2), vancomycin-resistant *S. aureus* (VRSA) strain NRS281 (CFS-255), and MSSA strain NRS77 (CFS-1155). The CLSI-recommended QC strain *S. aureus* ATCC 29213 is described as weak beta-lactamase *mecA* negative (13) was obtained from American Type Culture Collection (Manassas, VA). The MRSA strains CFS-2718 and CFS-2720 and MSSA strains CFS-2705 and CFS-2708 are clinical blood culture isolates (USA, 2017). The genome sequence of both ATCC 29213 and MW2 is available (14, 15). All strains were propagated from frozen stocks by incubation for 24 h at 37°C on BBL Trypticase soy blood agar (TSAB) plates (Becton, Dickinson and Company, Sparks, MD, USA). Exebacase was supplied as 10.64 mg/mL in a liquid carrier solution containing proprietary components (ContraFect Corporation, Yonkers, NY, USA). Vancomycin (catalog no. V8138, lot no. 0000132463) and oxacillin (catalog no. 28221, lot no. BCBJ1695V) were purchased from Sigma-Aldrich (St. Louis, MO, USA), and stock solutions were prepared as described (13).

### Broth microdilution susceptibility testing

Exebacase MICs and minimum bactericidal concentrations (MBCs) were determined by the BMD method using CAMHB-HSD media (11, 13, 16, 17), comprising BBL Mueller-Hinton II (cation-adjusted) broth (catalog no. 212322, lot 5257869; Becton Dickinson, Franklin Lakes, NJ, USA) with 25% (vol/vol) horse serum (catalog no. H1270, lot no. 21C414; Sigma-Aldrich, St. Louis, MO) and 0.5 mM dl-dithiothreitol (final concentration), pH 7.2–7.4 (catalog no. 646563, lot no. MKCMO454; Sigma-Aldrich). MIC endpoints were read as described in CLSI document M100-ed33 (12). Antibiotic MICs and MBCs were determined as described in CLSI documents M07-A11 (16) and M26-A (17), respectively, and in CAMHB-HSD.

### Time-kill assay

Time-kill kinetics were examined according to the method described in CLSI document M26-A (17). Bacteria were suspended in CAMHB-HSD at a final concentration of ~5 × 10^6^ CFU/mL and exposed to exebacase for 24 h at 35°C in ambient air without agitation. Exebacase was tested at 8, 4, 2, 1, 0.5, and 0.25 × MIC values, specific for each strain tested (see Table 1 for MICs); untreated (vehicle) control was included for each strain tested. Just prior to the addition of exebacase and at 30 min, 1, 2, 3, and 24 h thereafter, culture samples were removed, 10-fold serially diluted, and plated on TSAB plates. After 24 h incubation at 37°C, colonies were counted from the dilution(s) with the most suitable colony count (1–30 colonies present per spot). Based on these counts, CFU/mL was calculated and plotted as Log_10_ CFU/mL vs time to generate a time-killing curve. Given the dilution scheme, the limit of detection was 400 CFU/mL. All treatments were performed in duplicate, and the mean CFU/mL was calculated. Treatment of MRSA and MSSA strains, respectively, with vancomycin and oxacillin, was performed as above in CAMHB-HSD. For cultures demonstrating regrowth after initial CFU decreases, resulting colonies were passaged twice sequentially on a TSAB plates, and MICs were determined and compared to original MIC values.

**TABLE 1:** MIC and MBC values (µg/mL) of exebacase, oxacillin, and vancomycin against *S. aureus* strains used in this study

Bacterial strain (phenotype)	MIC/MBC (µg/mL) in CAMHB-HSD			MIC/MBC (µg/mL) in CAMHB *^a^*	
	Exebacase	Vancomycin	Oxacillin	Vancomycin	Oxacillin
ATCC 29213 (MSSA)	0.5/0.5	n.d./n.d.^*b*^	0.5/0.5	n.d./n.d.	0.5/0.5
CFS-2705 (MSSA)	0.5/1	n.d./n.d.	0.5/1	n.d./n.d.	0.5/0.5
CFS-2708 (MSSA)	1/1	n.d./n.d.	0.5/0.5	n.d./n.d.	0.5/1
MW2 (MRSA)	0.5/0.5	1/1	n.d./n.d.	1/1	n.d./n.d.
CFS-2718 (MRSA)	0.5/1	1/1	n.d./n.d.	1/1	n.d./n.d.
CFS-2720 (MRSA)	0.5/1	1/2	n.d./n.d.	1/1	n.d./n.d.

^*a*^ For the oxacillin MICs, CAMHB was supplemented with 2% NaCl.

^*b*^ n.d., not determined.

### Lytic assay

Overnight cultures of strain MW2 (MRSA) and strain ATCC 29213 (MSSA) grown in BD Bacto Tryptic soy broth (TSB; catalog no. 211825, lot no. 8087697, Becton, Dickenson and Co.) were diluted 1:100 in TSB and grown for 2.5 h at 37°C with aeration. The exponential phase cells were washed, concentrated 10-fold in 20 mM phosphate buffer (PB) pH 7.4, and stored at 4°C for use within 30 min. Exebacase stock was thawed at 25°C for 5 min, vortexed for 2–3 s, and diluted to prepare a 0.512 mg/mL working stock in 20 mM PB. From the working stock, 0.2 mL (containing ~1 × 10^9^ bacteria) was added to each well of the first column of a Falcon 96-well, flat bottom, clear, sterile, non-treated, individually wrapped micro-titer plate. Serial twofold dilutions were then performed into 0.1 mL of PB across wells of the *x*-axis until column 11 where, after removing 0.1 mL, instead of transferring to column 12, the sample was discarded. From the *S. aureus* culture in PB prepared above, 0.1 mL was pipetted into all wells of columns 1–12. Once completed, the wells of columns 1–11 contained a final exebacase concentration range of 256–0.25 µg/mL. Wells of column 12 contained buffer and cells only (untreated control). Samples were mixed, and the optical density at 600 nm (OD_600_) was followed for 20 min at room temperature in a SpectraMax M3 Multi-Mode Microplate Reader (Molecular Devices, San Jose, CA, USA). The OD_600_ values were plotted vs time (s) over the 20-min period. For clarity, only data for concentrations from 64 to 1 µg/mL is shown. Additionally, the minimum amount of exebacase (µg) needed to decrease the initial OD_600_ by 50% was used to determine specific activity (Units/mg of exebacase, ±standard deviation [SD]) as previously described (18, 19). Analysis of the exebacase mutant variants against *S. aureus* ATCC 29213 was performed as described for exebacase.

### Assay for growth and bacteriolysis in CAMHB-HSD

Overnight cultures of *S. aureus* ATCC 29213 grown in CAMHB-HSD were diluted 1:100 in CAMHB-HSD and grown at 37°C with aeration. At an OD_600_ of 0.1–0.2, the culture was split into two equal volumes. Exebacase was added to a final concentration of 8 × MIC (4 µg/mL) to one of the cultures, the remaining culture served as untreated control. Both treated and untreated cultures were then incubated at 37°C with aeration. At the indicated time-points, 1 mL aliquots were removed to determine OD_600_. Viable counts (CFU/mL) were determined by quantitative plating. In brief, 0.1 mL aliquots of both the control and treated cultures were added to the first row of a 96-well polypropylene microtiter plate. The culture samples were then serially diluted 10-fold across each of seven wells containing 90 µL of phosphate-buffered saline (PBS) each. After the dilution, 25 µL of each well (last 6 wells) was spotted on TSAB plates, which were then air-dried and incubated overnight at 35°C. The next day, plates were observed, and colonies were counted for the dilution(s) with the most suitable colony count (1–30 colonies present per spot) and CFU/mL was calculated (limit of detection was 40 CFU/mL). Culture samples were also removed just prior to and at the indicated times after the addition of exebacase for analysis by transmission electron microscopy (TEM), as described below.

### Exebacase active site mutants

The C26S and H102A mutant variants of exebacase were generated using PCR site-directed mutagenesis. Forward and reverse versions of the following oligonucleotides were designed to encode the desired amino acid substitutions: for C26S, (5′) GGTAACGGTGAAAGCTACGCTCTGGCTACTGGTACGAACG and for H102A, (5′) GTAACCCGTACGGCGCCGTTGTTATCGTTGAAG. The mutations were introduced into plasmid pBAD24-CF-301 (the exebacase open reading frame) using the above primers by PCR that amplified the entire plasmid. Parental template was removed from the PCR product using a methylation-dependent endonuclease (*Dpn*I), and the resulting products were agarose gel purified, ligated, and transformed into TOP10 *Escherichia coli* bacteria (ThermoFisher Scientific) on carbenicillin selective plates. Transformants were screened using either *Nhe*1 (C26S) or *Nar*1 (H102A), and positive clones were confirmed by DNA sequencing (Genewiz South Plainfield, NJ, USA). Purification of exebacase and the two mutant variants was performed for the lytic assay analysis, in the manner previously described (2, 20).

### Electron microscopic analysis of killing

An overnight culture of ATCC 29213 was diluted 1:100 in CAMHB-HSD and grown at 37°C with aeration to an OD_600_ of 0.1. The culture was then split into two equal volumes, one to serve as the untreated (vehicle) control and the other to which exebacase was added to a final concentration of 4 µg/mL (8 × MIC). Just prior to the addition of exebacase and at intervals thereafter up to 4 h, aliquots were removed from the treated and untreated cultures to determine both OD_600_ and viable cell numbers by quantitative plating on TSAB plates. Samples were also prepared for TEM by pelleting 1.5 mL of culture at 4,000 × *g* for 3 min at 15°C, resuspending in 25 mM 4-(2-hydroxyethyl)−1-piperazineethanesulfonic acid (HEPES) buffer (pH 7.4), mixing at a ratio of 1:1 with a solution of 4% paraformaldehyde and 5% glutaraldehyde in 0.1 M sodium cacodylate buffer (2× fixative), and incubating at room temperature for 30 min. The samples were next pelleted at 4,000 × *g* for 3 min at 15°C, resuspended in 2% paraformaldehyde and 2.5% glutaraldehyde in 0.1 M sodium cacodylate buffer (1× fixative), and stored at 4°C. At the Analytical Imaging Facility of Albert Einstein College of Medicine (New York), the samples were postfixed with 1% osmium tetroxide followed by 2% uranyl acetate, dehydrated through a graded series of ethanol, and embedded in Spurrs resin (Electron Microscopy Sciences, Hatfield, PA). Ultrathin sections were cut on a Leica Ultracut UC7, stained with uranyl acetate followed by lead citrate, and viewed on a JEOL 1400 Plus Transmission Electron Microscope at 120kv. The TEM analysis of killing for strain MW2 was performed in the same manner.

### Video microscopy

An overnight culture of MW2 was diluted 1:100 in TSB and grown for 2.5 h at 37°C with aeration. The exponential phase cells were washed, concentrated 10-fold in 20 mM phosphate buffer pH 7.4, and stained with 4′,6-diamidino-2-phenylindole, dihydrochloride (DAPI; catalog no. D1306, Thermo Fisher Scientific) according to the manufacturer’s protocol. Bacteria were analyzed using an ONIX Microfluidic Perfusion Platform (CellASIC) in a BO4A microfluidic bacteria plate. Flow chambers contained bacteria in a single focal plane and were first washed for 30 min at 37°C with PB using a flow rate of 2 pounds per square inch (psi). Chambers were exposed to PB with exebacase (1 µg/mL, 2 × MIC) for 1 h at 37°C using a flow rate of 2 psi. Bacteria were imaged using a Nikon Ti Live Imaging system (100× oil immersion lens) with exposure times of 40 ms. For the analysis of CFS-255, exponential cells were prepared and stained with DAPI as above, prior to treatment with exebacase (1 µg/mL) for 10 min in PB; fluorescence images were acquired (using Q Capture Pro software) in a Nikon Eclipse 80i fluorescence microscope equipped with a Nikon Plan Apo 100X oil immersion objective, and the resulting video was condensed to 20 s. *S*train CFS-255 was likewise treated with exebacase (1 µg/mL, 2 × MIC) for 10 min in PB and examined by light microscopy in the Nikon Eclipse 80i fluorescence microscope; the resulting video was condensed to 7 s.

### Visual analysis of cidality

Overnight cultures of MW2 and CFS-1155 were diluted 1:100 in TSB and grown at 37°C with aeration. At OD_600_ 1.0 (~5 × 10^8^ CFU/mL), 0.8 mL was transferred into Fisherbrand Disposable Cuvettes (1.5 mL semi-micro cell, polystyrene, catalog no. 14–955-127). For treated cultures, exebacase was added to final concentration of 64 µg/mL. For untreated cultures, PBS alone was added. Cultures were immediately visualized using an MP4 video format on a Nikon Coolpix B500 camera set at 1080 resolution, 30 FPS, 29 min (maximal time allowed). The resulting video was cropped, and playback speed was adjusted to cover the first 15 min in a 14-s video with resolution set to 720 p.

## RESULTS

### MIC and MBC determinations

The MIC and MBC data for exebacase, oxacillin (MSSA only), and vancomycin (MRSA only) are presented in Table 1. The MIC and MBC values for each antibiotic determined in CAMHB were equivalent to the activity observed in CAMHB-HSD, as previously described (7). MBC values were within twofold of the MIC values for all entities, consistent with cidal activity of exebacase for each of the isolates.

### Killing kinetics for MSSA and MRSA

**Fig 1:**
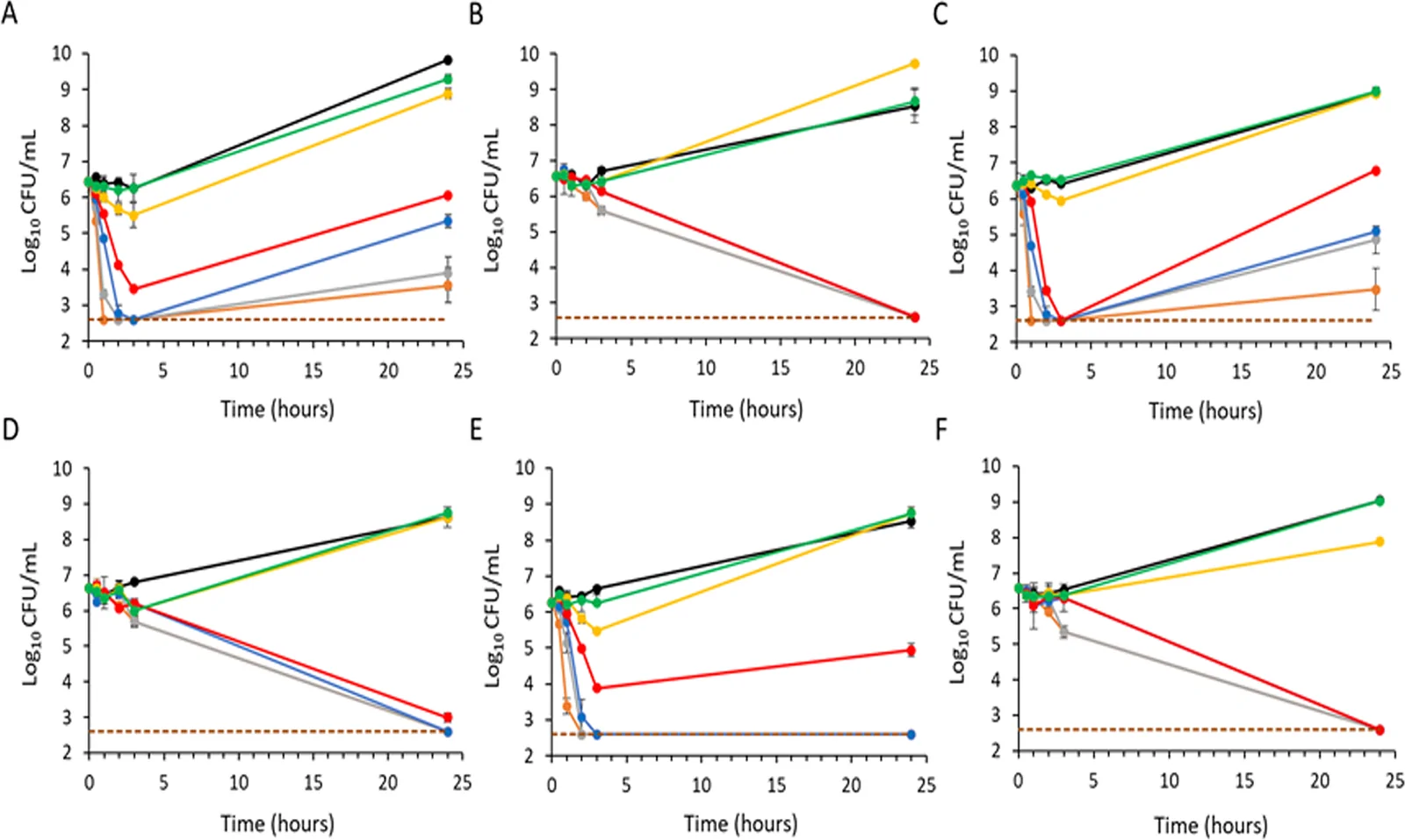
Time-kill curves of exebacase and vancomycin against MRSA in CAMHB-HSD media. Strains MW2, CFS-2718, and CFS-2720 were treated with exebacase (**A**, **C**, and **E**, respectively) or vancomycin (**B**, D, and **F**, respectively). Treatment groups include the following: black, vehicle control; green, 0.25 × MIC; yellow, 0.5 × MIC; red, 1 × MIC; blue, 2 × MIC; gray, 4 × MIC; and orange, 8 × MIC. For each strain, the exebacase MIC is 0.5 µg/mL, and the vancomycin MIC is 1 µg/mL. Analyses were performed in duplicate, and mean values are indicated (±standard deviation).

**Fig 2:**
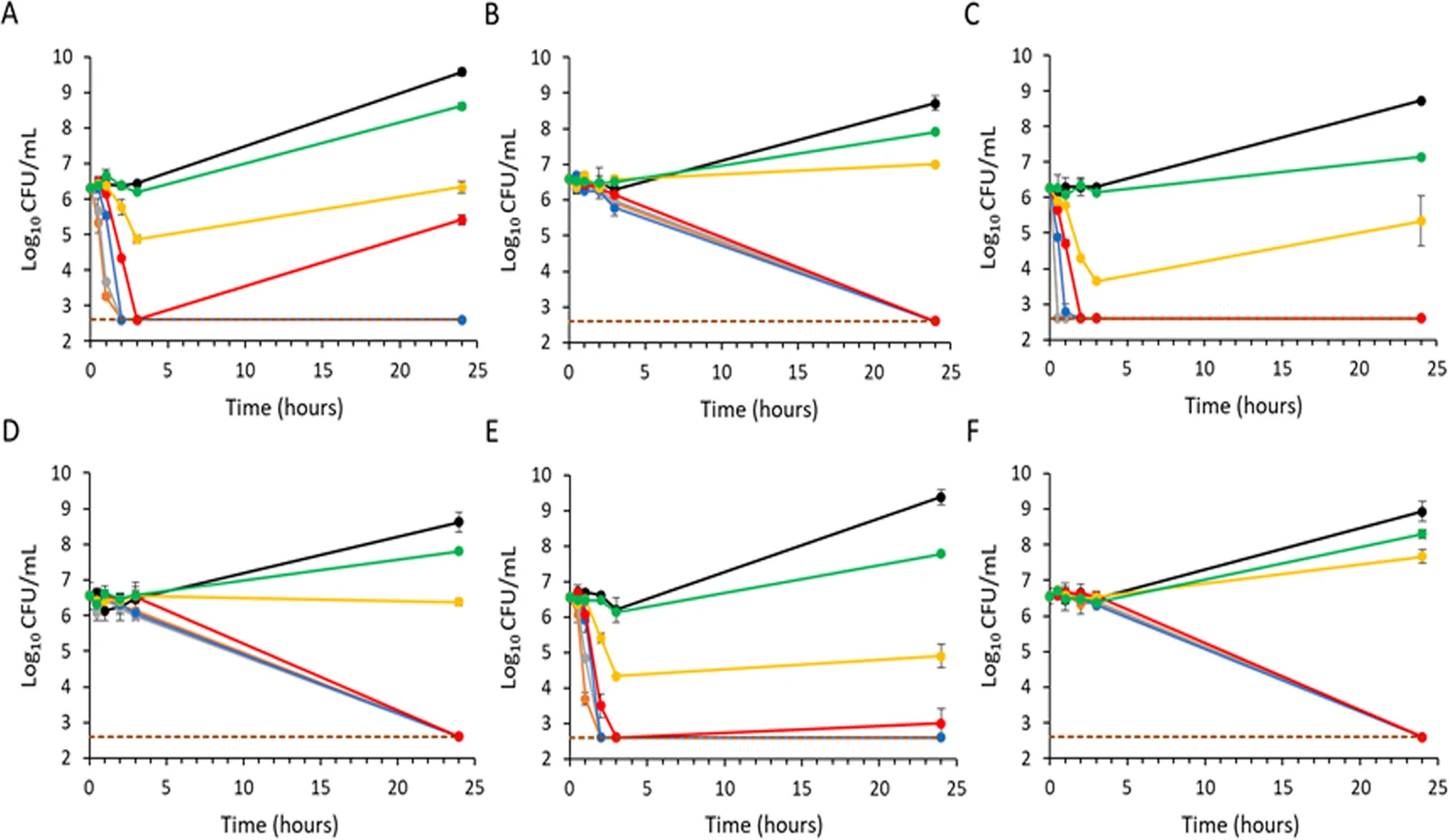
Time-kill curves of exebacase and oxacillin against MSSA in CAMHB-HSD media. Strains ATCC 29213, CFS-2705, and CFS-2708 treated with exebacase (**A**, **C**, and **E**, respectively) and oxacillin (**B**, **D**, and **F**, respectively). Treatment groups include the following: black, vehicle control; green, 0.25 × MIC; yellow, 0.5 × MIC; red, 1 × MIC; blue, 2 × MIC; gray, 4 × MIC; and orange, 8 × MIC. For each strain, the exebacase MIC is 0.5 µg/mL, and the oxacillin MIC is 0.5 µg/mL. Analyses were performed in duplicate, and mean values are indicated (±standard deviation).

Time-kill assays with exebacase against 3 MRSA (Fig. 1A, C, and E) and 3 MSSA (Fig. 2A, C, and E) isolates showed a concentration-dependent bactericidal effect with a 3-log_10_ CFU/mL reduction within 2 h of exposure at 1–2 × MIC or higher, with some regrowth by 24 h.

Regrowth following a bactericidal effect in the time-kill assay format is commonly observed with lysins (21) and is believed to arise from rapid “overkilling,” whereby cellular debris encases some staphylococci and provides a protective effect that later enables regrowth as lysin concentrations decline through binding to cellular debris. This effect is likely limited to *in vitro* conditions (21). Following CLSI recommendations for regrowth observed in time-kills (17), exebacase MICs were determined for survivors and shown to be identical to that of isolates prior to treatment; thus, survival was not be attributed to resistance. Furthermore, no change in MBC values for the survivors was observed.

In contrast to the rapid killing observed with exebacase, both vancomycin against MRSA isolates and oxacillin against MSSA isolates showed a slower time-dependent killing (Fig. 1B, D, and F and Fig. 2B, D, and F, respectively), with only about 1-log_10_ CFU/mL killing activity in 3 h. Consistent with the slower killing, no regrowth was observed.

### Rapid loss of optical density in exebacase-treated cultures

**Fig 3:**
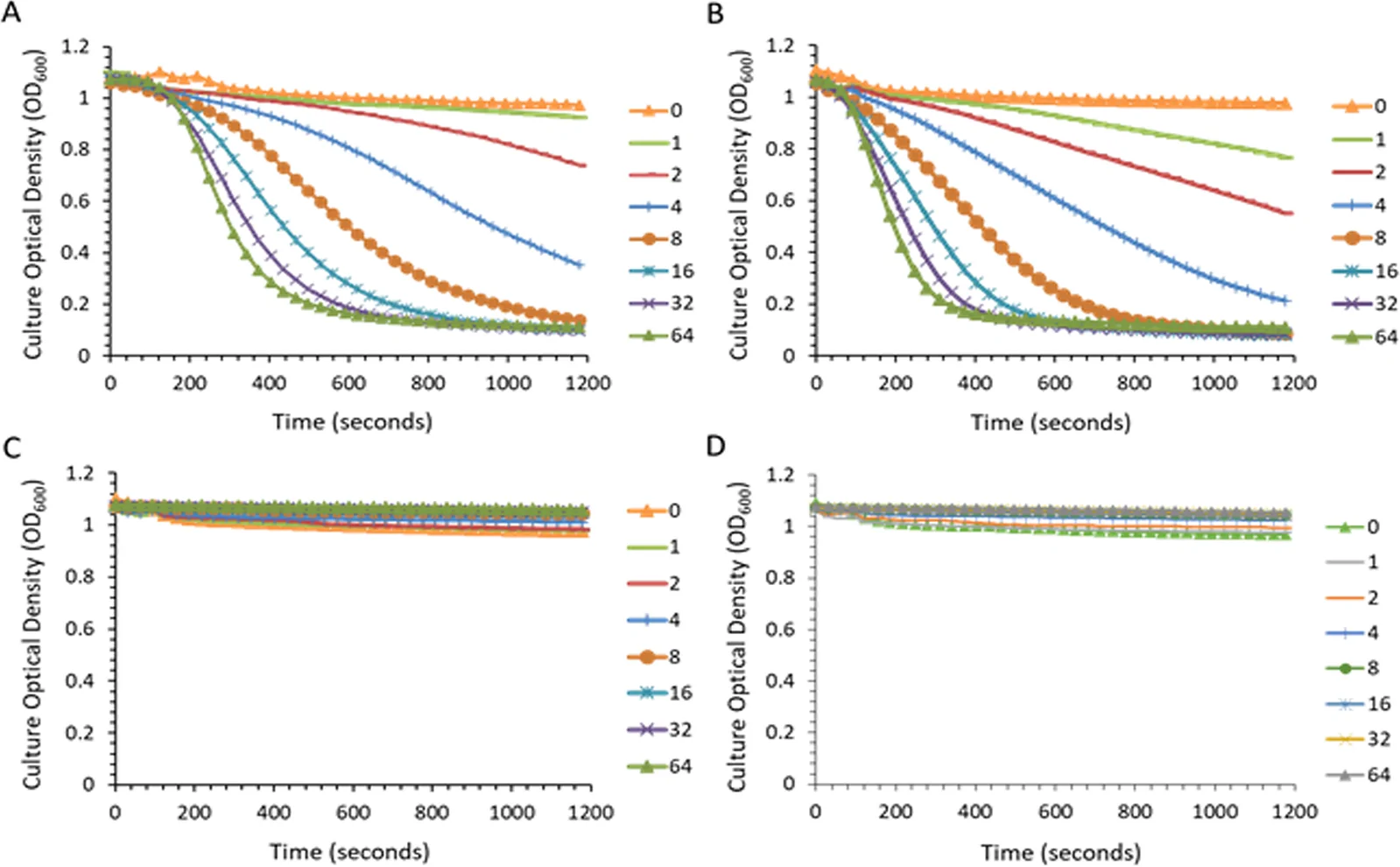
Turbidity reduction (lytic) assay of exebacase activity. Strains MW2 (**A**) and ATCC 29213 (**B**) were treated with a range of exebacase concentrations (values in µg/mL). Strain ATCC 29213 treated with enzymatically inactive exebacase variants, C26S (**C**) and H102A (**D**), over a range of concentrations (values in µg/mL).

The lytic assay is a spectrophotometric-based method that follows reduction in turbidity (i.e., loss of OD_600_) in a standardized bacterial suspension added to a lysin dilution series to quantitate enzymatic (lytic) activity against the target pathogen (22). Using this method, concentration-dependent bacteriolytic effects were observed for both MW2 and ATCC 29213 treated with exebacase over a 20-min period (Fig. 3A and B, respectively). A minimum concentration of 4 µg/mL was required to elicit a 50% decrease in the initial OD_600_ of both strains. Specific activity values of 2,789 (SD = ±392) and 3,328 (SD = ±28.1) units/mg of exebacase were determined for MW2 and ATCC 29,213, respectively.

Based on findings that exebacase is an endopeptidase and a member of the cysteine-histidine-dependent amidohydrolase/peptidase family (2, 19, 23, 24), two putative active site residues were disrupted and the resulting mutants (C26S and H102A) were studied as inactive controls. Consistent with inactivation, the exebacase MIC values for both the C26S and H102A variants were >512 µg/mL against both MW2 and ATCC 29213. Likewise, the mutants were inactive in the lytic assay as shown for ATCC 29213 in Fig. 3C and D, with specific activity values of <40 units/mg. These findings confirm that the enzymatic activity of exebacase is required for bacteriolysis.

### Loss of optical density and viability is concurrent with bacteriolysis

**Fig 4:**
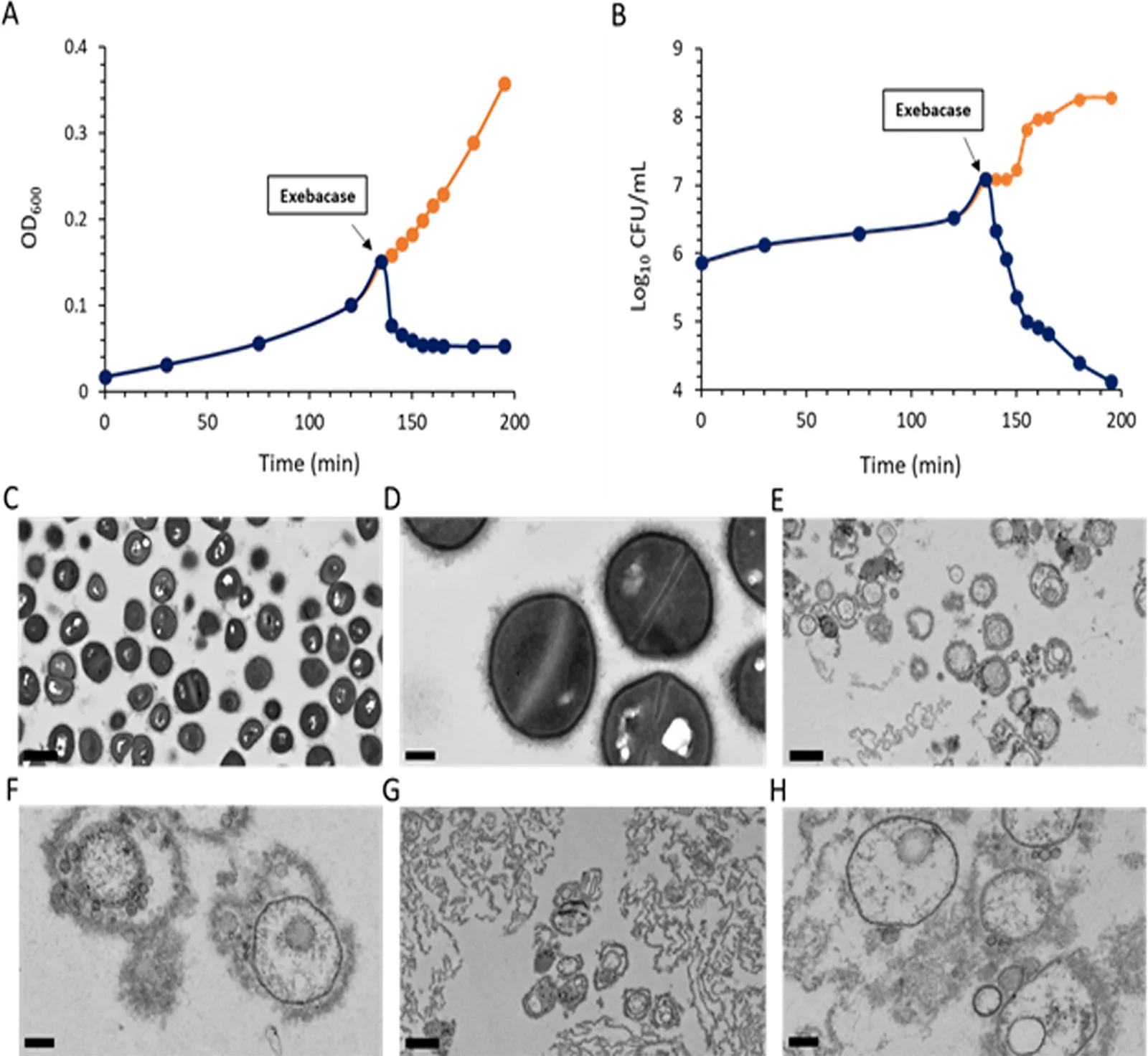
Impact of exebacase treatment on the optical density, viability, and ultrastructure of ATCC 29213 (MSSA) grown in CAMHB-HSD. (**A**) Culture OD_600_ was monitored before and after the indicated addition of exebacase at 8 × MIC (4 µg/mL) (blue); untreated control was included (orange). (**B**) Bacterial viability (CFU/mL) before and after the indicated addition of exebacase at 8 × MIC (4 µg/mL) (blue); untreated control was included (orange). (**C and D**) TEM analysis of culture sample just prior to the addition of exebacase (viewed at 2,500× and 10,000× magnification, respectively). (**E and F**) TEM analysis of culture sample after 5 min of exposure to exebacase (viewed at 2,500× and 10,000× magnification, respectively). (**G and H**) TEM analysis of culture sample after 60 min of exposure to exebacase (viewed at 2,500× and 10,000× magnification, respectively). Scale bars are 1 µm and 200 nm for the 2,500× and 10,000× magnifications, respectively.

Addition of exebacase to an exponential phase culture of ATCC 29213 at 8 × MIC (4 µg/mL) resulted in an immediate reduction in OD_600_ and viability (CFU/mL) (Fig. 4A and B). Culture samples examined by TEM just prior to the addition to exebacase revealed a population of intact cells with well-defined cell envelope structures (Fig. 4C and D). However, within 5 min of the addition of exebacase, and concurrent with loss of OD_600_ and viability, bacteriolysis was evident, marked by the appearance of cellular debris and bacterial ghost cells which, while lacking cytoplasmic material, contained bilayered membrane-like spherical blebs (Fig. 4E and F). By 60 min, the exebacase treatment resulted primarily in bacterial debris (Fig. 4G and H).

**Fig 5:**
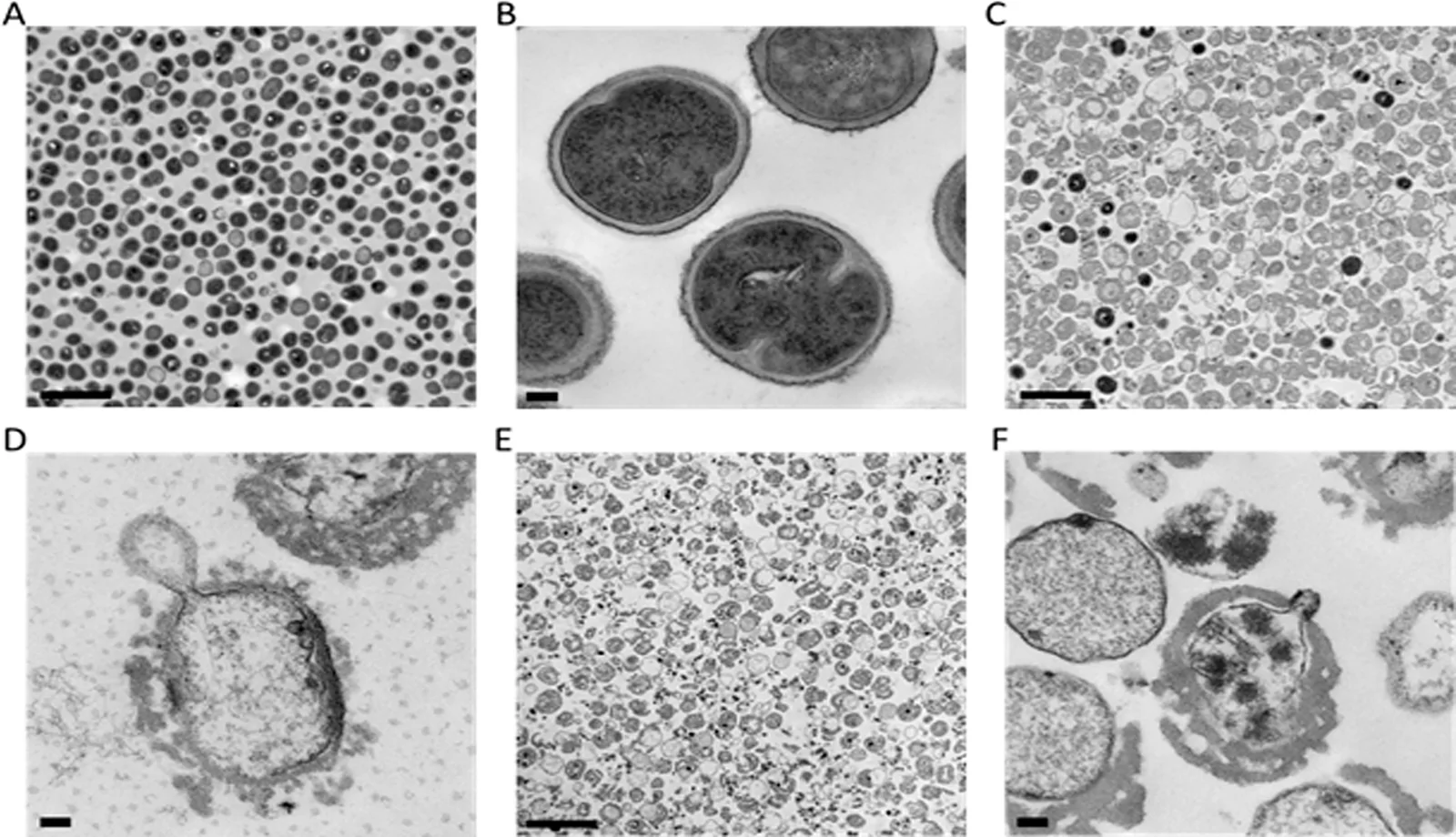
Transmission electron micrographs of MW2 (MRSA) before and after treatment with exebacase at 8 × MIC (4 µg/mL). Viable cells just prior to the addition of exebacase were viewed at 13,300× (**A**) and 114,000× (**B**) magnification (scale bars 2 µm and 100 nm, respectively). Bacteria at 3 min post treatment were viewed at 13,300× (**C**) and 114,000× (**D**) magnification (scale bars 2 µm and 100 nm, respectively). Bacteria at 30 min post treatment viewed at 13,300× (**E**) and 114,000× (**F**) magnification (scale bars 2 µm and 100 nm, respectively).

The impact of exebacase treatment on MW2 was likewise examined. For a uniform population of healthy coccoid cells (Fig. 5A and B), treatment with exebacase at 8 × MIC (4 µg/mL) for just 3 min resulted in widespread bacteriolysis, marked by cellular debris and bacterial ghost cells lacking cytoplasmic material (Fig. 5C and D). By 30 min, no cells with intact electron dense cytoplasmic material were observed (Fig. 5E and F).

The rapid reductions in turbidity of exebacase-treated cultures of MW2 and NRS77 were visually demonstrated with video microscopy over a 15-min period (condensed into 14 s videos) upon addition of exebacase (Videos S1 and S2, respectively). Using fluorescence microscopy, bacteriolysis of both MW2 and NRS-281 was demonstrated by the rapid loss of DAPI-stained DNA material within 10 min after the addition of exebacase (Video S3 and S4). For NRS-281, bacteriolysis (over a 10-min period) was also examined by light microscopy, which demonstrated regions of localized cell wall expansion, likely associated with extrusion of cytoplasmic material (Video S5).

## DISCUSSION

Lysins are peptidoglycan hydrolases that degrade the mesh-like cell wall serving to protect bacteria from internal turgor pressure that can reach up to 20 atmospheres in Gram-positive organisms including *S. aureus* (25). The capacity of recombinantly produced and purified lysins to kill target microorganisms in a rapid process of “lysis from without” serves as the basis for potential therapeutic use of the lysin class of antimicrobial agents (1).

For exebacase, a lysin in clinical development, an understanding of the killing kinetics provides important qualitative information on the pharmacodynamics and mechanism of action. The current study used CAMHB-HSD, a newly developed media that determines the activity of exebacase more accurately (11, 12), in the time-kill assay format to investigate kill kinetics for exebacase against a range of *S. aureus* strains including MRSA, MSSA, and VRSA phenotypes. Bactericidal reductions in viability of 3-log_10_ CFU/mL were observed for all strains tested within 2 h following exposure to exebacase. The rapid concentration-dependent bactericidal effect of exebacase was in contrast to the time-dependent cidal activity of vancomycin and oxacillin, two conventional antibiotics that interfere with peptidoglycan biosynthesis, and which showed only about 1-log_10_ CFU/mL bactericidal reduction over 3 h, consistent with other reports (26, 27). Regrowth at 24 h was observed with exebacase for all isolates in the time-kill assays, a reported phenomenon with lysins (21). This regrowth is likely due to the very rapid mode of action for lysins, wherein large amounts of simultaneously released cellular debris enclose occasional surviving staphylococci, which are protected from the lysin and subsequently allow for regrowth.

The lytic assay provided an independent examination of killing in both CAMHB-HSD and buffer conditions, wherein rapid reductions in optical density, consistent with bacteriolysis and coincident with a loss of bacterial viability, were observed within minutes after the addition of exebacase. Likewise, by TEM, only 3 min were required for bacteriolysis which was marked by membrane destabilization and loss of electron dense cytoplasmic material (explaining the loss of optical density observed in the lytic assay); remaining material included bacterial ghosts (cell envelope lacking cytoplasmic material) and cellular debris. The rapid kill effect was, thus, similar across all three methods tested, including the time-kills performed in AST media, the lytic assay performed in buffer, and the lytic assay/CFU loss assay performed in AST media for the microscopy study. Any subtle difference may be attributed to the differences in the conditions used.

These findings are consistent with a killing effect upon contact similar to that with other lysins (22, 28). While the exact basis of killing remains to be elucidated, it is most likely that the enzymatic activity of exebacase (i.e., cleavage of peptidoglycan) causes localized regions of membrane destabilization and, concomitant, release of cytoplasmic material. The rapid loss of DAPI fluorescence observed with labeled staphylococci that occurred almost immediately after the addition of exebacase supports this hypothesis and taken with the brightfield analysis suggests that rapid expansions of wall material occur concomitantly with the forceable ejection of nucleoid material.

The rapid-kill property associated with lysins makes them well suited to quickly reduce the bacterial burden in infected hosts and kill target microorganisms in a manner distinct from but complementary to conventional antibiotics. Antibiotics are certainly powerful drugs that can kill or inhibit the growth of bacteria; however, the exposure times required for such activities also do enable bacteria to evolve and evade their effects of through multiple different mechanisms. Lysins, alternatively, exert their direct lytic effects on contact, through the engagement of surface-exposed peptidoglycan (as is the case with Gram-positive organisms) and the cleavage of bonds which are not necessarily amenable to alterations and which are required to withstand the internal osmotic pressure; this activity explains the rapid nature of killing and perhaps the low propensity for resistance developed observed for lysins. As such, the combination of lysins to kill or destabilize large bacterial populations with antibiotics to leverage synergy is a very promising antimicrobial strategy in an era requiring such novel solutions.

## SUPPLEMENTAL MATERIAL

Legends spectrum.01906-23-s0001.pdf Legends to Videos S1 to S5.

Video S1 spectrum.01906-23-s0002.mp4 Exponential phase MRSA strain MW2 suspended at a concentration of ~1x10e8 CFU/mL in 1 ml polypropylene cuvettes and treated with either buffer (vehicle control; on left) or exebacase (32 µg/mL; on right) for 15 min. The resulting video is condensed into 14 sec.

Video S2 spectrum.01906-23-s0003.mp4 Exponential phase MSSA strain CFS 1155 suspended at a concentration of ~1x10e8 CFU/mL in TSB (using 1 ml polypropylene cuvettes) and treated with exebacase (32 µg/mL) for 15 min. The resulting video is condensed into 14 sec.

Video S3 spectrum.01906-23-s0004.mp4 MRSA strain MW2 (labeled with DAPI) suspended in Phosphate Buffer was treated with exebacase (1 µg/mL) for 2 h and fluorescence images were taken every 2 min. The resulting video was condensed into 18 sec.

Video S4 spectrum.01906-23-s0005.mp4 VRSA strain CFS-255 (NRS 281) labeled with DAPI and suspended in Phosphate Buffer was treated with exebacase (1 µg/mL) for 10 min. The resulting video was condensed into 19 sec.

Video S5 spectrum.01906-23-s0006.mp4 VRSA strain CFS-255 (NRS 281) suspended in phosphate buffer and treated with exebacase (1 µg/mL) for 10 min. The resulting video was condensed into 7 sec.

ASM does not own the copyrights to Supplemental Material that may be linked to, or accessed through, an article. The authors have granted ASM a non-exclusive, world-wide license to publish the Supplemental Material files. Please contact the corresponding author directly for reuse.

## References

[1] Schuch R , Cassino C , Vila-Farres X . 2022. Direct lytic agents: novel, rapidly acting potential antimicrobial treatment modalities for systemic use in the era of rising antibiotic resistance. Front Microbiol 13:841905. doi:10.3389/fmicb.2022.841905 35308352 PMC8928733

[2] Schuch R , Lee HM , Schneider BC , Sauve KL , Law C , Khan BK , Rotolo JA , Horiuchi Y , Couto DE , Raz A , Fischetti VA , Huang DB , Nowinski RC , Wittekind M . 2014. Combination therapy with lysin CF-301 and antibiotic is superior to antibiotic alone for treating methicillin-resistant Staphylococcus aureus-induced murine bacteremia. J Infect Dis 209:1469–1478. doi:10.1093/infdis/jit637 24286983 PMC3982849

[3] Schuch R , Khan BK , Raz A , Rotolo JA , Wittekind M . 2017. Bacteriophage lysin CF-301, a potent antistaphylococcal biofilm agent. Antimicrob Agents Chemother 61:e02666-16. doi:10.1128/AAC.02666-16 28461319 PMC5487678

[4] Kebriaei R , Stamper KC , Lev KL , Morrisette T , Abdul-Mutakabbir JC , Schuch R , Lehoux D , Rybak MJ . 2021. Exebacase in addition to daptomycin against MRSA. Antimicrob Agents Chemother 65:e0012821. doi:10.1128/AAC.00128-21 34398668 PMC8522749

[5] Karau M , Schmidt-Malan S , Mandrekar J , Lehoux D , Schuch R , Cassino C , Patel R . 2022. Locally delivered antistaphylococcal lysin exebacase or CF-296 is active in methicillin-resistant Staphylococcus aureus implant-associated osteomyelitis. J Bone Jt Infect 7:169–175. doi:10.5194/jbji-7-169-2022 36032801 PMC9399932

[6] Mendes RE , Lindley J , Gurung N , Castanheira M , Castanheira M , Schuch R , Ambler JE . 2021. In vitro activity of exebacase (CF-301) against Staphylococcus aureus causing bacteremia in the United States, including multidrug-resistant subsets, abstr P-61. Open Forum Infect Dis 8:S621–S622. doi:10.1093/ofid/ofab466.1253

[7] Watson A , Sauve K , Cassino C , Schuch R . 2020. Exebacase demonstrates in vitro synergy with a broad range of antibiotics against both methicillin-resistant and methicillin-susceptible Staphylococcus aureus. Antimicrob Agents Chemother 64:e01885-19. doi:10.1128/AAC.01885-19 31712212 PMC6985718

[8] Oh JT , Cassino C , Schuch R . 2019. Postantibiotic and sub-MIC effects of exebacase (lysin CF-301) enhance antimicrobial activity against Staphylococcus aureus. Antimicrob Agents Chemother 63:e02616-18. doi:10.1128/AAC.02616-18 30936103 PMC6535547

[9] Murray E , Draper LA , Ross RP , Hill C . 2021. The advantages and challenges of using endolysins in a clinical setting. Viruses 13:680. doi:10.3390/v13040680 33920965 PMC8071259

[10] Fischetti VA , Nelson D , Schuch R . 2006. Reinventing phage therapy: are the parts greater than the sum? Nat Biotechnol 24:1508–1511. doi:10.1038/nbt1206-1508 17160051

[11] Oh JT , Ambler JE , Cassino C , Schuch R . 2021. Development of a broth microdilution method for exebacase susceptibility testing. Antimicrob Agents Chemother 65:e0258720. doi:10.1128/AAC.02587-20 33903102 PMC8218677

[12] CLSI . 2023. Performance standards for antimicrobial susceptibility testing, document M100-33rd edition. Clinical and Laboratory Standards Institute, Wayne, PA.

[13] CLSI . 2020. Performance standards for antimicrobial susceptibility testing, document M100 - 30th Ed. Clinical and Laboratory Standards Institute, Wayne, PA.

[14] Soni I , Chakrapani H , Chopra S . 2015. Draft genome sequence of methicillin-sensitive Staphylococcus aureus ATCC 29213. Genome Announc 3. doi:10.1128/genomeA.01095-15 PMC4582586 26404610

[15] Baba T , Bae T , Schneewind O , Takeuchi F , Hiramatsu K . 2008. Genome sequence of Staphylococcus aureus strain Newman and comparative analysis of staphylococcal genomes: polymorphism and evolution of two major pathogenicity islands. J Bacteriol 190:300–310. doi:10.1128/JB.01000-07 17951380 PMC2223734

[16] CLSI . 2018. Methods for dilution antimicrobial susceptibility tests for bacteria that grow aerobically, document M07 - 11th Ed. Clinical and Laboratory Standards Institute, Wayne, PA.

[17] CLSI . 1999. Methods for determining bactericidal activity of antimicrobial agents; approved guideline, document M-26A. Clinical and Laboratory Standards Institute, Wayne, PA.

[18] Indiani C , Sauve K , Raz A , Abdelhady W , Xiong YQ , Cassino C , Bayer AS , Schuch R . 2019. The antistaphylococcal lysin, CF-301, activates key host factors in human blood to potentiate methicillin-resistant Staphylococcus aureus bacteriolysis. Antimicrob Agents Chemother 63:e02291-18. doi:10.1128/AAC.02291-18 30670427 PMC6437495

[19] Lood R , Molina H , Fischetti VA . 2017. Determining bacteriophage endopeptidase activity using either fluorophore-quencher labeled peptides combined with liquid chromatography-mass spectrometry (LC-MS) or forster resonance energy transfer (FRET) assays. PLoS One 12:e0173919. doi:10.1371/journal.pone.0173919 28296948 PMC5352010

[20] Gilmer DB , Schmitz JE , Euler CW , Fischetti VA . 2013. Novel bacteriophage lysin with broad lytic activity protects against mixed infection by Streptococcus pyogenes and methicillin-resistant Staphylococcus aureus. Antimicrob Agents Chemother 57:2743–2750. doi:10.1128/AAC.02526-12 23571534 PMC3716137

[21] Kaspar U , Schleimer N , Idelevich EA , Molinaro S , Becker K . 2022. Exploration of bacterial re-growth as in vitro phenomenon affecting methods for analysis of the antimicrobial activity of chimeric bacteriophage endolysins. Microorganisms 10:445. doi:10.3390/microorganisms10020445 35208898 PMC8877451

[22] Schuch R , Nelson D , Fischetti VA . 2002. A bacteriolytic agent that detects and kills Bacillus anthracis. Nature 418:884–889. doi:10.1038/nature01026 12192412

[23] Lu JZ , Fujiwara T , Komatsuzawa H , Sugai M , Sakon J . 2006. Cell wall-targeting domain of glycylglycine endopeptidase distinguishes among peptidoglycan cross-bridges. J Biol Chem 281:549–558. doi:10.1074/jbc.M509691200 16257954

[24] Mitkowski P , Jagielska E , Nowak E , Bujnicki JM , Stefaniak F , Niedziałek D , Bochtler M , Sabała I . 2019. Structural bases of peptidoglycan recognition by lysostaphin SH3b domain. Sci Rep 9:5965. doi:10.1038/s41598-019-42435-z 30979923 PMC6461655

[25] Whatmore AM , Reed RH . 1990. Determination of turgor pressure in Bacillus subtilis: a possible role for K^+^ in turgor regulation. J Gen Microbiol 136:2521–2526. doi:10.1099/00221287-136-12-2521 2127801

[26] McKay GA , Beaulieu S , Arhin FF , Belley A , Sarmiento I , Parr T , Moeck G . 2009. Time-kill kinetics of oritavancin and comparator agents against Staphylococcus aureus, Enterococcus faecalis and Enterococcus faecium. J Antimicrob Chemother 63:1191–1199. doi:10.1093/jac/dkp126 19369269

[27] Woods GL , Yam P . 1988. Bactericidal activity of oxacillin against beta-lactamase-hyperproducing Staphylococcus aureus. Antimicrob Agents Chemother 32:1614–1618. doi:10.1128/AAC.32.11.1614 3266987 PMC175938

[28] Nelson D , Loomis L , Fischetti VA . 2001. Prevention and elimination of upper respiratory colonization of mice by group a Streptococci by using a bacteriophage lytic enzyme. Proc Natl Acad Sci U S A 98:4107–4112. doi:10.1073/pnas.061038398 11259652 PMC31187

