# Supplementary material for: Rapid bacteriolysis of Staphylococcus aureus by lysin exebacase
Source: Microbiol Spectr. 2023 Aug 10;11(5):e01906-23. doi: 10.1128/spectrum.01906-23 (PMC10580946; doi:10.1128/spectrum.01906-23)
Supplement: Legends — to Videos S1 to S5. [file spectrum.01906-23-s0001.pdf]

Supplementary Video File Legend

Video S1. Exponential phase MRSA strain MW2 suspended at a concentration of ~$1x10^8$ CFU/mL in 1 ml polypropylene cuvettes and treated with either buffer (vehicle control; on left) or exebacase (32 µg/mL; on right) for 15 min. The resulting video is condensed into 14 sec.

Video S2. Exponential phase MSSA strain CFS 1155 suspended at a concentration of ~$1x10^8$ CFU/mL in TSB (using 1 ml polypropylene cuvettes) and treated with exebacase (32 µg/mL) for 15 min. The resulting video is condensed into 14 sec.

Video S3. MRSA strain MW2 (labeled with DAPI) suspended in Phosphate Buffer was treated with exebacase (1 µg/mL) for 2 h and fluorescence images were taken every 2 min. The resulting video was condensed into 18 sec.

Video S4. VRSA strain CFS-255 (NRS 281) labeled with DAPI and suspended in Phosphate Buffer was treated with exebacase (1 µg/mL) for 10 min. The resulting video was condensed into 19 sec.

Video S5. VRSA strain CFS-255 (NRS 281) suspended in Phosphate Buffer and treated with exebacase (1 µg/mL) for 10 min. The resulting video was condensed into 7 sec.
